# Multilevel and geo-statistical modeling of malaria risk in children of Burkina Faso

**DOI:** 10.1186/1756-3305-7-350

**Published:** 2014-07-29

**Authors:** Sekou Samadoulougou, Mathieu Maheu-Giroux, Fati Kirakoya-Samadoulougou, Mathilde De Keukeleire, Marcia C Castro, Annie Robert

**Affiliations:** Pôle Epidémiologie et Biostatistique (EPID), Institut de Recherche Expérimentale et Clinique (IREC), Faculté de Santé Publique (FSP), Université catholique de Louvain (UCL), Clos Chapelle-aux-champs 30, bte B1.30.13, 1200 Bruxelles, Belgium; Department of Global Health & Population, Harvard School of Public Health, Boston, MA USA; Georges Lemaitre Center for Earth and Climate Research, Earth and Life Institute (ELI), Université catholique de Louvain (UCL), Louvain-la-Neuve, Belgium

**Keywords:** Burkina Faso, *Plasmodium falciparum*, Geo-statistics, Random effect models, Bayesian variable selection, Malaria burden, Integrated Nested Laplace Approximation

## Abstract

**Background:**

Previous research on determinants of malaria in Burkina Faso has largely focused on individual risk factors. Malaria risk, however, is also shaped by community, health system, and climatic/environmental characteristics. The aims of this study were: i) to identify such individual, household, community, and climatic/environmental risk factors for malaria in children under five years of age, and ii) to produce a parasitaemia risk map of Burkina Faso.

**Methods:**

The 2010 Demographic and Health Survey (DHS) was the first in Burkina Faso that tested children for malaria parasitaemia. Multilevel and geo-statistical models were used to explore determinants of malaria using this nationally representative database.

**Results:**

Parasitaemia was collected from 6,102 children, of which 66.0% (95% confidence interval (CI): 64.0-68.0%) were positive for *Plasmodium spp*. Older children (>23 months) were more likely to be parasitaemic than younger ones, while children from wealthier households and whose mother had higher education were at a lower risk. At the community level, living in a district with a rate of attendance to health facilities lower than 2 visits per year was significantly associated with greater odds of being infected. Malaria prevalence was also associated with higher normalized difference vegetation index, lower average monthly rainfall, and lower population densities. Predicted malaria parasitaemia was spatially variable with locations falling within an 11%-92% prevalence range. The number of parasitaemic children was nonetheless concentrated in areas of high population density, albeit malaria risk was notably higher in the sparsely populated rural areas.

**Conclusion:**

Malaria prevalence in Burkina Faso is considerably higher than in neighbouring countries. Our spatially-explicit population-based estimates of malaria risk and infected number of children could be used by local decision-makers to identify priority areas where control efforts should be enhanced.

**Electronic supplementary material:**

The online version of this article (doi:10.1186/1756-3305-7-350) contains supplementary material, which is available to authorized users.

## Résumé

**Introduction:**

La recherche sur les déterminants du paludisme au Burkina Faso a permis d’identifier de nombreux facteurs liés aux caractéristiques individuelles. Cependant, le rôle des facteurs contextuels caractérisant l’ensemble des personnes d’un même ménage ou d’une même communauté, qui utilisent le même système de santé et qui vivent dans les mêmes conditions climatiques et environnementales a largement été ignoré. Les objectifs de cette étude chez les enfants âgés de moins de cinq ans étaient donc i) d’identifier les déterminants de la parasitémie palustre qui sont d’ordre individuels, ceux qui sont liés au ménage, ceux qui sont d’ordre communautaire, et ceux qui sont d’ordre climatique/environnemental et ii) de cartographier la prévalence de parasitémie palustre pour l’ensemble du Burkina Faso.

**Méthodes:**

La quatrième Enquête Démographique et de Santé (DHS) réalisée en 2010 était la première enquête dans laquelle les enfants ont été testés pour les infections à *Plasmodium sp*. au Burkina Faso. A partir de cette enquête représentative au niveau national, des modèles multiniveaux et géostatistiques ont été utilisés pour explorer les déterminants du paludisme.

**Résultats:**

Un total de 6,102 enfants ont été testés pour le paludisme et 66.0% (intervalle de confiance à 95%: 64.0-68.0%) d’entre eux étaient infectés par *Plasmodium spp*. Les plus âgés (>23 mois) avaient une parasitémie plus élevée. Les enfants des ménages ayant un statut socio-économique plus élevé et ceux dont la mère avait une scolarisation plus longue avaient une parasitémie plus faible. Au niveau communautaire, les enfants résidant dans les régions administratives où le taux de fréquentation des centres de santé n’atteignait pas deux visites par an étaient significativement plus à risque d’être parasités. Le risque de paludisme était également plus élevé quand l’indice de végétation normalisé était plus élevé, quand la moyenne des précipitations mensuelles était plus basse et quand la densité de population était plus faible. Les prédictions de la prévalence du paludisme variaient selon les localités, allant de 11% à 92%. Le nombre d’enfants ayant la parasitémie palustre est concentré dans les zones à haute densité de population alors que le risque parasitémique est plus élevé dans les zones rurales peu peuplées.

**Conclusion:**

La prévalence du paludisme au Burkina Faso est considérablement plus élevée que celle des pays voisins, mais il existe une forte variabilité à l’intérieur du pays. Nos estimations du risque de parasitémie palustre et du nombre d’enfants infectés devraient permettre aux décideurs locaux d’identifier les zones d’intervention prioritaires pour le renforcement de la prévention et du contrôle de la maladie.

## Background

In 2010, there were an estimated 216 million cases of malaria and an estimated 660,000 malaria deaths worldwide [1], with children under five years being the most affected. There are, however, major regional variations in the burden of malaria, with highest prevalences observed in sub-Saharan countries like Burkina Faso [1]. The costs of malaria to individuals, families, and communities are considerable in this landlocked country. In fact, malaria represents the leading cause of medical consultation in Burkina Faso. Among children under five years of age, malaria accounted for 61.4% of medical consultations, 77.7% of hospitalizations, and was responsible for almost 80% of deaths in 2011 [2]. These figures provide an incomplete estimate of the total malaria burden in this country because many Burkinabé lack proper access to health care services and are therefore not accounted for in the statistics presented above.

According to the 2013 World Malaria Report, the entire population of Burkina Faso is at high risk of malaria [3]. Coverage of insecticide-treated nets (ITNs) has been estimated at 57% of households in Burkina Faso and less than half of children aged less than five years are sleeping under an ITN [4]. Although an artemisinin-based combination therapy (ACT) policy has been adopted by Burkina Faso as first-line treatment against malaria in 2005 [5], these treatments are not yet free of charge for children. In the country, district-specific statistics suggest that there are considerable variations in malaria prevalence because of different ecological conditions, variations in health system performance, and socioeconomic differentials or intervention coverage. Efficient interventions and preventive efforts could be improved by furthering our understanding of geographic prevalence patterns and the factors underlying them. Previous studies conducted in Burkina Faso have described potential determinants of malaria at the individual level but contextual factors have been suggested to play an important role [6, 7]. Yet, few studies have addressed the impact of such factors that could operate at a broader scale and play a role in the performance of preventive programs. For example, environmental factors such as rainfall, altitude and temperature, that affect *Anopheles sp*. bionomics, are associated with malaria transmission in endemic countries [8–11].

Although Burkina Faso is considered as one of the most highly malaria-endemic countries in Africa [1], geographic variability in disease prevalence within this country is poorly understood. Bayesian geo-statistical models have been successfully used to map malaria risk in a number of countries [12–16] and could be useful to target interventions in regions most at need. Previously published prevalence data for Burkina Faso are of limited value because they were extrapolated from convenience samples or from non-random sentinel populations. Demographic and Health Surveys (DHSs) provide nationally- and regionally-representative data on socio-demographic characteristics and on the burden of malaria prevalence and its determinants [4]. From April 2010 to January 2011, Burkina Faso performed its fourth DHS, and it included for the first time a malaria parasitaemia test among children under five years of age. This DHS offers a unique opportunity to determine geographic patterns of malaria and to explore determinants of prevalence among children. The goals of this study were thus: i) to examine individual, household, community characteristics and climatic/environmental factors associated with malaria infection, and ii) to produce a high-resolution map of parasitaemia risk in Burkina Faso in order to improve control efforts.

## Methods

### Study area

Burkina Faso is a landlocked country of 274,200 km^2^ located in West Africa: it shares borders with Ivory Coast, Ghana, Togo and Benin in the South, Mali in the North and Niger in the North-West. The total population is estimated at 17 million. Most of central Burkina Faso is on a savanna plateau while the south is green and the north is arid.

Burkina Faso has a tropical climate with two seasons: the country receives between 600 and 900 millimeters of rainfall during the rainy season and a hot dry wind from the Sahara blows during the dry season. The rainy season lasts approximately four months, July to October, and is shorter in the north of the country. Three climatic zones can be defined: the Sahel, the Sudan-Sahel, and the Sudan-Guinea. The Sahel in the north typically receives less than 600 millimeters [17] of rainfall per year and has high temperatures that can reach up to 47°C. The Sudan-Sahel region is a transitional zone with regards to rainfall and temperature. Further to the south, the Sudan-Guinea zone receives more than 900 millimeters [17] of rain each year and has cooler average temperatures. This is why the duration of the high malaria transmission season, which is the rainy season, varies slightly from the northern to the southern part of the country. The main malaria vectors are *An. gambiae* s.s., *An. arabiensis*, and to a lesser extent, *An. funestus*[18]*.* The predominant malaria parasite is *Plasmodium falciparum*, which accounts for more than 95% of infections in children under five years of age [19].

### Demographic and health survey data

Children under five years of age were sampled from a total of 572 clusters in the 2010 Burkina Faso DHS. Clusters were defined as “Zones de dénombrement”: the smallest administrative unit with an average population of 1,000 and of 1,200 in rural and in urban areas, respectively. Our study sample included all 6,102 children. The DHS is a national household-based survey conducted by the National Center for Statistics and Demography (NCSD) and Macro International in order to assess disease and risk factors among the population. The survey had a stratified two-stage design. A total of 574 clusters were selected at the first stage with a proportional-to-population-size-probability sample. At the second-stage, a complete listing of all households was carried out and households were randomly selected for inclusion in the survey with equal probability. Data were collected between April 2010 and January 2011.

Blood samples were drawn and interviews were conducted in the selected households (50% of households were selected for malaria testing). All children aged between 6 and 60 months living in these households were eligible for malaria testing. Parents or guardians provided consent for their children’s participation (non-consent was 1%). The parasite prevalence survey was based on microscopic examinations of stained blood films. Two blood slides, each composed of thick and thin films, were taken from each participant. Blood slides were read by two technicians at a reference laboratory in Ouagadougou (i.e., the *Centre National de Recherche sur le Paludisme*) and classified qualitatively as either negative or positive infection. The presence of *Plasmodium sp.* parasites in blood films was the primary outcome of this study. Detailed information on the survey’s design and laboratory procedures can be found in the Burkina Faso 2010 DHS report [4].

### Potential determinants of malaria risk

Individual-level variables included: age (categorized as six-12 months, 13–23 months, 24–35 months, 36–47 months and 48–59 months), gender and a binary variable indicating if children slept under a bed net the night before the survey. At the household level, we considered: mother’s education; household size reporting the number of children aged under five years living in the household; the wealth index, based on ownership of household assets, used as a proxy for socio-economic status (SES) [20]. The wealth index was divided into quintiles, corresponding to the poorest to the richest.

At the community level, we considered a number of district-specific variables such as: rate of attendance to health facilities, the population per medical doctor ratio, and the population per health worker ratio. This information was extracted from the Ministry of Health Statistical Yearbook of 2010 [2] which is an annual compilation of reports and information gathered from all the 63 health districts in the country's healthcare system in 2010. These indicators were geographically linked to the DHS data by matching the centroid of the DHS cluster to a map of health districts.

Environmental conditions can impact on malaria transmission and we examined the effect of a number of climatic and environmental variables: distance from rivers and water bodies, distance from major roads, normalized-difference vegetation index (NDVI, a proxy for moisture), altitude, minimum temperature, maximum temperature, mean diurnal range in temperature, rainfall before the survey date, average monthly rainfall, precipitation seasonality, vegetation cover, and population density (i.e., a proxy for the rural–urban gradient). These climatic and environmental variables were assembled from a variety of sources summarized in Table 1. The geographic coordinates of the DHS clusters’ centroid were used to link the climatic/environmental variables to the parasitaemia survey (for raster-based data, we assigned to each cluster the characteristics of the pixel that corresponded to its geographic location). All geospatial data manipulations were performed using the geographic information system (GIS) software QGIS version 2.0 [21].

**Table 1:** **Overview of data sources for the climatic and environmental variables**

Variables	Time period (Resolution)	Spatial resolution	Source
Minimum temperature*	Average of 1950-2000	~1 km	Global Climate Data http://www.worldclim.org
Average temperature			
Maximum temperature*			
Mean diurnal range in temperature			
Average monthly rainfall			
Precipitation seasonality			
Altitude	2000		
NDVI^†^	2001-2010 (5 days)	250 m	US Geological Survey http://earlywarning.usgs.gov/
Rainfall before the survey	2010 (10 days)	~8 km	
MODIS VCF^§^	2010	~250 m	Global Land Cover Facility http://glcf.umd.edu/data/vcf/
Distance to roads^††^	1998	Vector	DIVA-GIS http://www.diva-gis.org/
Distance to water bodies^††^	1998	Vector	*Food and Agriculture Organization* http://www.fao.org/geonetwork/srv/en/main.home
Population density	2010	~5 km	Gridded Population of the World (v3) http://sedac.ciesin.columbia.edu/data/collection/gpw-v3

Unless stated otherwise, the variables described above were linked to the Demographic and Health Survey (DHS) records by assigning them the information corresponding to the geographic location of the DHS clusters’ centroid.

*Averages computed for minimum temperatures of the coldest month and maximum temperatures of the warmest month.

^†^NDVI: Normalized-Difference Vegetation Index.

^§^VCF: Vegetation Continuous Field.

^††^Distance to roads and water bodies were obtained by calculating the Euclidian distance from the DHS cluster’ centroid to the closest road and water body.

### Statistical analyses

#### Multilevel analysis

Descriptive statistics were used to examine the characteristics of the sample and, unless stated otherwise, sampling weights were not used in the statistical analyses. Given the hierarchical structure of the data, logistic random effect models were used in univariate and multivariate analyses to identify factors associated with malaria infection. Potential non-linear relationships between covariates and the malaria parasitaemia outcome were accounted for by either categorizing the covariate or including polynomial terms. The appropriate functional form was chosen by visually inspecting outcome-covariate scatter plots and through consideration of the Deviance Information Criterion (DIC) [22]. Specifically, a two-level model was specified with a binary response *Y*_*ij*_, indicating if child *i* living in community *j* was found to be parasitaemic. The logistic regression model had the following form:


This generalized linear model has a fixed part (β_0_ + βX_ij_) estimating the conditional vector of coefficients β = (β_1_, …, β_k_)^T^ for the matrix of explanatory covariates (X_ij_), a random intercept attributable to communities (ω_*j*_), and a random error for child *i* that doesn’t depend on community (ϵ_*i*_). Note that preliminary analyses suggested that the inclusion of a household-level random effect was not required as its variance was extremely small. Statistical analyses were performed in a Bayesian framework and the marginal posterior distributions of the parameters were estimated using Integrated Nested Laplace Approximations (INLA) [23].

In order to build parsimonious and well identifiable models, Bayesian variable selection was performed [22, 24, 25]. Specifically, we used a Gibbs variable selection algorithm [26] that used spike and slab priors on the model’s coefficients [27] (see Additional file 1: Text S1 for details). Results are presented for the univariate analyses and for the reduced multivariate model that includes only the covariates from the Bayesian variable selection.

#### Bayesian geo-statistical model

The models described above do not consider the spatial relationships among community locations. The standard way of incorporating geographical dependence in the model is by introducing spatially correlated random effects at every sampled location, generally assuming that the spatial correlation decreases as distances between location increases. Predictions for un-sampled locations are also improved by explicitly modeling spatial dependence. Two models were fitted: model A that included individuals, household, community, and climatic/environmental variables, and model B that included the climatic and environmental variables. Producing a risk map of malaria requires that we have complete spatial information over the whole geographic area of Burkina Faso (at both sampled and unsampled locations) for all covariates included in the geo-statistical model. Because such information was unavailable in unsampled locations for SES, bednet usage, mother’s education, etc., only model B could be used for prediction. These geo-statistical models have the following general form:


where ω_j_ ~ *N*(0,Σ) with element 

Here, *β*_*0*_, *βX*_*ij*_, and *ϵ*_*i*_ are the same as described before but ω_j_ is now the spatial random effect for community *j*. Σ_*kl*_ represents the Matérn covariance between location *k* and *l*. It was assumed that this random effect has a multivariate normal distribution with mean zero. The covariance is a function of the variance of the spatial process (σ^2^), a scaling parameter (κ), the Euclidean distance between location *k* and *l* (*d*_*kl*_), a smoothness parameter (ν) fixed to one in this particular application, and Κ_ν_ and Γ(ν) are the modified Bessel function of second kind order and the Gamma function, respectively. Further, the range of the spatial process is defined to be , which is approximately the distance to which the covariance function becomes negligible (i.e., <0.1).

The relationships between climatic and environmental variables with malaria parasitaemia were visually examined to determine the appropriate functional form. Further, the Deviance Information Criterion (DIC) was used to choose among linear, categorical or polynomial form for each of the variables. Because of potential biologically determined lag between climatic variables and malaria transmission, the DIC was used to choose the appropriate lag time among a number of plausible lags ranging from 30 to 70 days for rainfall and 7 to 21 days for NDVI. Based on these preliminary analyses, the rainfall estimates were lagged by 60 days and NDVI by 7 days. The Gibbs variable selection algorithm described in the preceding section was used to select a parsimonious set of climatic/environmental variables for inclusion (called model B in the Results section). These variables could be correlated with individual, household, or community characteristics and we wanted to give precedence to the covariates for which complete spatial coverage was available. Bayesian variable selection was therefore carried out in two stages. First, we considered only the set of 13 climatic/environmental variables for inclusion (Stage 1). Second, based on the reduced set of covariates from stage 1, we performed variable selection on an additional set of 9 individual-, household-, and community-level covariates while adjusting for the climatic/environmental variables selected during the first stage (Stage 2).

The out-of-sample predictive ability of our model was assessed by fitting the model to a random sample of 80% of the locations (432 clusters) and predicting the remaining 20% (108 clusters). We calculated the posterior predictive distributions of the test locations and computed the proportion of the observed parasitaemia prevalence falling into different coverage distributions (e.g. 10% to 100%). The mean predictive error and the squared root of the mean square error (RMSE) between the observed and predicted cluster prevalence were also calculated as an index of accuracy. The Area Under the Curve (AUC) of the Receiver Operating Characteristic (ROC) was used as an additional tool to assess model performance [28].

The computational burden of the geo-statistical models was overcome by fitting the model using INLA [23], which provides fast and accurate calculations of posterior marginal distributions [29]. Model parameters were estimated using the INLA library in R [30] and vague priors were used for all hyperparameters and parameters (see Additional file 1: Text S1 for details). Malaria risk and the estimated number of infected children for the entire country of Burkina Faso were mapped at a resolution of 2.5 km (44,206 unique locations) for the month of August 2010. This arbitrarily chosen reference period corresponds to the rainy season, which last from July to October in most of Burkina Faso.

### Ethical approval

Informed consent was provided by all survey participants (or their guardian) before questionnaire administration and malaria testing. Further, all survey protocols have been approved by the Internal Review Board of ICF International in Calverton (USA) and by the “Comité National d'Éthique” in Burkina Faso.

## Results

### Sample characteristics

The total number of households selected for the survey was 4,029 in 572 communities, and microscopy data were available for 6,102 children. A total of 34 communities, totaling 361 children, were missing geographical coordinates and had to be dropped from our analyses. The mean age of children was 32.3 months (range: 6–59 months). Overall, 83.6% of the children lived in rural areas, 50.8% were boys, and most mothers had no education (84.4%). The average household size was 7.8 persons. Most of the data (62.3%) were collected between July and October 2010 but all data from Central Region (5.9%) were taken between May-June 2010. The ratio of population per medical doctor was higher than 20,000 for all communities, and only 13.5% of children had more than two contacts with health facilities in 2010. The weighted prevalence of malaria in Burkina Faso was estimated at 66.0% (95% CI: 63.1-66.7) ranging for 27.7% in the Central Region to 77.7% in the Sud Ouest Region (Additional file 2: Figure S1).

### Multilevel analysis

Results of the multilevel logistic analyses are presented in Table 2. The unadjusted odds ratio (OR) multilevel analyses indicated that all predictors (except sex and population density) were significantly associated with malaria infection. Regarding individual-level variables, children who didn’t sleep under a bed net the night before the survey and children above 23 months of age had higher malaria prevalence. At the household level, having a mother with no schooling significantly increased the odds of being infected with malaria. On the other hand, living in areas with a high population density (i.e., urban), in a household with less than 5 individuals, and having a greater wealth index were associated with lower odds of malaria parasitaemia. Moreover, living in a community with a health facility rate of attendance greater than two visits per year, a population/doctor ratio between 20,000 and 50,000, and a population/health worker ratio below 3,000 were associated with lower odds of malaria infection among children.

**Table 2:** **Factors associated with malaria infection in univariate and multivariate random effect logistic regression analyses for children under five years of age in Burkina Faso (2010)**

Covariates	Univariate multilevel	Multivariate multilevel
	OR* (95% CrI ^†^ )	OR* (95% CrI ^†^ )
**Individual level factors**		
**Age** (6–12 months)	Reference	Reference
13-23 months	1.13 (0.91-1.40)	1.11 (0.90-1.37)
24-35 months	**1.46 (1.18-1.92)**	**1.41 (1.14-1.74)**
36-47 months	**1.71 (1.38-2.13)**	**1.70 (1.37-2.10)**
48-59 months	**1.92 (1.54-2.40)**	**1.81 (1.45-2.25)**
**Female Gender**	1.12 (0.99-1.27)	NI
**Not sleeping under a bed net**	**1.23 (1.08-1.41)**	NI
**Household level factors**		
**Mother’s Education Level** ^§^ (No schooling)	Reference	Reference
Primary	**0.54 (0.45-0.66)**	**0.73 (0.59-0.89)**
Secondary or higher	**0.30 (0.22-0.40)**	**0.60 (0.44-0.82)**
**Household size** (<5 persons)	Reference	
5-7 persons	1.18 (0.99-1.40)	NI
>7 persons	**1.50 (1.26-1.78)**	NI
**Household wealth index** (Richest)	Reference	Reference
Richer	**3.05 (2.46-3.79)**	**1.84 (1.45-2.33)**
Middle	**4.41 (3.50-5.54)**	**2.35 (1.82-3.05)**
Poorer	**4.42 (3.49-5.59)**	**2.23 (1.70-2.90)**
Poorest	**5.68 (4.46-7.25)**	**3.02 (2.29-3.99)**
**Community level factors**		
**Rate of attendance to health facilities** (>2)	Reference	Reference
1-2 visits per year	**2.49 (1.81-3.40)**	**1.38 (1.05-1.82)**
<1 visits per year	**6.13 (4.07-9.21)**	**2.49 (1.77-3.51)**
**Population per medical doctor** (20,000-50,000)	Reference	
50,001–100,000	**2.32 (1.65-3.26)**	NI
>100 000	**2.76 (1.94-3.91)**	NI
**Population per heath worker** (>3,000)	**1.84 (1.46-2.33)**	NI
**Climatic/Environmental factors**		
**Distance to river** (<1 km)	Reference	
1 - 5 km	0.99 (0.52-1.87)	NI
≥5 km	**2.00 (1.10-3.62)**	NI
**Distance to road**: truncated at 16 km	**1.32 (1.22-1.43)**	NI
Distance to road: squared term	**0.99 (0.98-0.99**)	NI
**NDVI** (<0.3)	Reference	Reference
0.3 - 0.5	**1.82 (1.40-2.37)**	**1.34 (1.07-1.68)**
≥0.5	**2.77 (2.06-3.73)**	**1.71 (1.29-2.26)**
**Average monthly rainfall** (<50 mm)	Reference	
50 -100 mm	**0.49 (0.31-0.79)**	NI
≥100 mm	**0.32 (0.19-0.56)**	NI
**Rainfall 60 days prior to survey** (<100 mm)	**4.06 (2.54-6.47)**	**1.69 (1.14-2.53)**
**Altitude** (<300 m)	**1.82 (1.43-2.30)**	
**Maximum temperature (**<38.5°C)	Reference	Reference
38.5 - 39.5°C	**0.72 (0.55-0.94)**	0.89 (0.70-1.12)
≥39.5°C	1.09 (0.78-1.51)	0.89 (0.67-1.19)
**Population density**: 100/km^2^, truncated at 500/km^2^	0.89 (0.57-1.37)	1.35 (0.88-2.06)
Population density: squared term	0.94 (0.87-1.02)	**0.91 (0.84-0.99)**

Statistically significant results are in bold.

*OR: Odds Ratio using the category between brackets as reference.

^†^CrI: Credible Interval.

^§^210 observations were missing mother’s education information and these were removed from the models that included this variable.

NI: Not Included.

After performing Bayesian variable selection on these covariates (Table 2), the multivariate results showed that individual/household-level variables that significantly reduced the odds of having malaria were: age of the child, mother’s education, and household wealth index. Different community-level covariates reduced the odds of individual parasitaemia: higher attendance rate to health facilities, NDVI, rainfall in previous 60 days before the survey, maximum temperature, and population density.

### Geo-statistical analysis

The variables selected for inclusion in the final geo-statistical model are presented in Table 3. The posterior model probability of the ‘best’ climatic/environmental model was 3.2% - rather weak at the first stage but increasing to 40.9% when performing Bayesian variable selection on the individual and community covariates and after adjusting for the climatic/environmental variables at the second stage of the multilevel modeling.

**Table 3:** **Parameter estimates from the geo-statistical models of malaria prevalence in children under five years of age in Burkina Faso (2010)**

Variables	Model A	Model B
	OR* (95% CrI ^†^ )	OR* (95% CrI ^†^ )
**Individual level factor**		
**Age** (6–12 months)	Reference	
13-23 months	1.10 (0.89-1.36)	
24-35 months	**1.41 (1.14-1.74)**	
36-47 months	**1.70 (1.37-2.10)**	
48-59 months	**1.79 (1.44-2.22)**	
**Household level factors**		
**Mother’s Education Level** ^§^ (No schooling)	Reference	
Primary	**0.75 (0.62-0.92)**	
Secondary or higher	**0.58 (0.42-0.78)**	
**Household wealth index** (Richest)	Referent	
Richer	**1.77 (1.40-2.24)**	
Middle	**2.24 (1.73-2.90)**	
Poorer	**2.10 (1.61-2.73)**	
Poorest	**2.76 (2.09-3.64)**	
**Rate of attendance to health facilities** (>2)	Reference	
1-2 visits per year	**1.36 (1.01-1.83)**	
< 1 visit per year	**2.21 (1.56-3.13)**	
**Climatic/Environmental factors**		
**NDVI (<0.3)**	Reference	Reference
0.3 - 0.5	**1.23 (1.00-1.53)**	**1.28 (1.03-1.60)**
≥0.5	**1.66 (1.27-2.17)**	**1.87 (1.42-2.46)**
**Average monthly rainfall** (<50 mm)	Reference	Reference
50 - 100 mm	0.69 (0.45-1.05)	**0.55 (0.36-0.84)**
≥100 mm	**0.44 (0.26-0.76)**	**0.33 (0.19-0.58)**
**Rainfall 60 days prior to survey** (<100 mm)	**1.75 (1.18-2.58)**	**1.97 (1.33-2.91)**
**Maximum temperature (**<38.5°C)	Reference	Reference
38.5 - 39.5°C	**0.74 (0.56-0.98)**	**0.73 (0.54-0.97)**
≥39.5°C	**0.66 (0.47-0.93)**	0.73 (0.51-1.01)
**Population density**: 100/km^2^, truncated at 500/km^2^	1.25 (0.82-1.90)	1.10 (0.72-1.71)
Population density: squared term	0.93 (0.86-1.01)	0.94 (0.87-1.02)
	**Mean (95% CrI**†**)**	**Mean (95% CrI**†**)**
**Spatial variance**	0.48 (0.28-0.83)	0.63 (0.40-1.03)
**Range of the spatial effect (km)**	8.8 (4.9-15.8)	8.9 (5.2-14.6)

Statistically significant results are in bold.

*OR: Odds Ratio using the category between brackets as reference.

^†^CrI: Credible Intervals.

^§^210 observations were missing mother’s education information and these were removed from model A.

**Figure 1:**
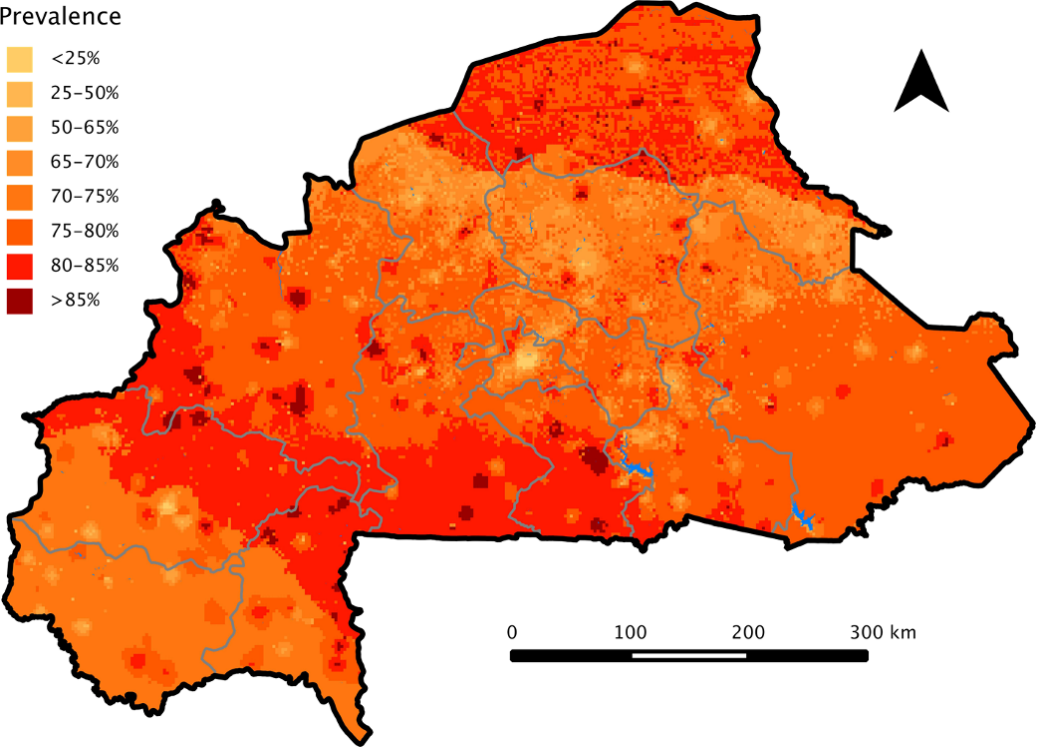
**Geostatistical map of malaria parasitaemia risk in children under 5 years in Burkina Faso for August 2010.**

Results from final model A (Table 3) demonstrated that age, mother’s education, wealth, rate of attendance to health facilities, NDVI, rainfall before the survey, average monthly rainfall, and maximum temperature were independently associated with malaria after accounting for spatial dependence. The geo-statistical models also indicated that malaria risk can be highly variable over relatively short distances, with spatial variances of 0.48-0.63 and an estimated spatial range of roughly 9 km. Models A and B both had an AUC of 0.78, suggesting an acceptable/good predictive performance. Model B was used to predict malaria risk in children under five years at 44,206 unique locations. The malaria risk map produced for Burkina Faso is presented in Figure 1 and the maps of the posterior predictive distribution of the 2.5^th^ and 97.5^th^ percentiles are presented as supplementary online material (Additional file 3: Figure S2). Prevalence was very high (>60%) over vast areas of the country with a median predicted malaria prevalence of 77% (range of 11% to 92%). Areas of low risk were concentrated in major urban centers such as the capital Ouagadougou. The north of the country and the horizontal band between the cities of Bobo-Dioulasso and Ouagadougou were areas with the highest malaria risk.

**Figure 2:**
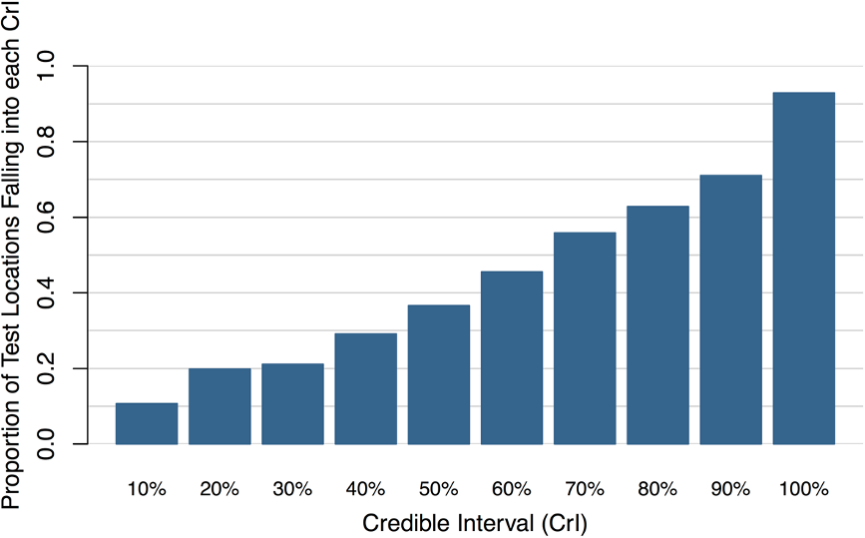
**Proportion of observed prevalence at test locations falling into different coverage of the posterior predictive distribution.**

Further, using 108 clusters as test locations (20%), the climatic/environmental (model B) geo-statistical model’s predictions had a mean error of 0.005 and a RMSE of 0.19. This out-of-sample validation shows that predictions were fairly accurate, with 77% of tested locations falling within the 95% credible intervals (CrI) of the posterior predictive distribution. The proportions of test locations falling into specific CrI coverage of the predictive posterior distributions are presented in Figure 2.

**Figure 3:**
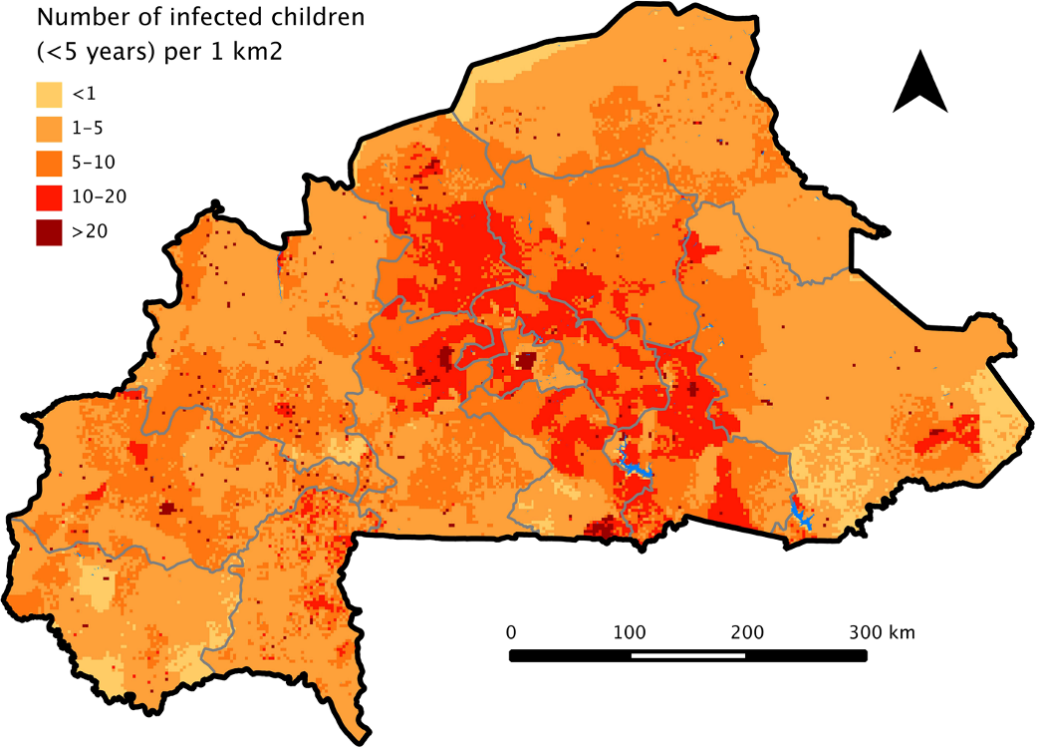
**Geostatistical map of the estimated number of Bukinabé children under five that are infected with malaria in 2010.**

Taking population density into account, the mean predicted malaria prevalence estimated from the geo-statistical model (model B) was 65%, a figure almost identical to that estimated from the DHS data alone. The map of the estimated number of infected children under 5 years showed that malaria burden was concentrated in major cities and in the districts around Ouagadougou (Figure 3). Thus, although malaria risk was higher in rural areas, malaria burden was concentrated in high population density districts.

## Discussion

In the present study, we identified determinants of malaria using the first nationally representative survey of malaria parasitaemia in Burkina Faso. Model-based infection risk maps for 2010 were produced and the number of infected children was estimated. Mapping malaria risk is now considered an important tool for sub-national targeting of intervention for malaria control [31]. We found that the burden of malaria among children continues to be extremely high, with two-thirds of children infected with *Plasmodium* parasites in Burkina Faso. Prevalence of malaria was higher than the one reported from other sub-Saharan African countries where comparable nationally representative data are available (Table 4). Although differences between the timing of data collection with respect to seasons of peak malaria transmission somewhat hampers our ability to compare these surveys, Benin and Ivory Coast had prevalence 2–4 times lower than the one recorded in Burkina Faso despite sharing national boundaries with Burkina Faso. Because of relatively similar climatic and/or environmental conditions, we attribute these country differences to factors like season of data collection, intervention coverage of malaria interventions and/or performance of health systems. For example, community rate of attendance at health facilities was shown to be an important determinant of malaria transmission in Burkina Faso. Interventions aimed at strengthening delivery of and access to health services could impact malaria transmission as coverage of core interventions such as insecticide-treated net, intermittent-preventive therapy, and provision of artemisinin-based combination therapies (ACT) are currently far below targets in Burkina Faso [32].

**Table 4:** **Prevalence of malaria (by microscopy) in children under five years from recent Demographic Health Surveys in sub-Saharan African countries**

Country	Survey dates	Rainy season*	Malaria prevalence (%)
Benin	12/2011 to 03/2012	April-July & October-November	28.4
Ivory Coast	12/2011 to 05/2012	April-July & October-November	18.0
Gambia	02/2013 to 04/2013	June-Octoberber	0.8
Ghana	09/2011 to 12/2011	April-July & September-October	27.5
Guinea	06/2012 to 10/2012	May-October	43.9
Liberia	09/2011 to 12/2011	May-October	27.8
Mali	11/2012 to 02/2013	June-October	51.6
Nigeria	10/2010 to 12/2010	April-October	42.0
Senegal	10/2010 to 04/2011	June-October	2.9
Tanzania	12/2011 to 05/2012	January-April & November-December	4.1
Uganda	11/2009 to 01/2010	March-May & September-November	44.7
Burkina Faso	05/2010 to 01/2011	July-October	66.0

*Because of sometime important within country variations in the timing and duration of the rainy season, this climatic indicator is reported for the capital region of each country.

Interestingly, rainfall was negatively associated with parasitaemia. This runs in opposition to a number of studies that reported that increased precipitation is associated with malaria transmission in Burkina Faso [19, 33, 34]. The survey used in this paper was, however, primarily conducted during the rainy season and malaria transmission often peaks at the beginning and at the end of this season. This association is believed to result from the negative impact of high precipitations on *Anopheles sp.* larval populations [35]. Our findings suggest that malaria is strongly associated with mother’s education. This finding was also observed in the 2010 Zambia National Malaria Indicator Survey [36]. We hypothesize that maternal education is positively associated with preventive care, which in turn is negatively associated with malaria risk in children under five. Indeed, Engle *et al.*[37] found that higher maternal education improves the mother’s ability to process information, acquire skills, and model behavior for their children.

With recent advances in geo-statistical modeling, spatially-explicit studies analyzing the relationship between disease status and ecological factors have given new insights into the epidemiology of malaria [10, 15, 38]. The accurate geographical identification and enumeration of individuals at risk is an important component of control efforts, facilitating better resource allocation, health management, and targeting interventions to maximize risk reduction.

The malaria risk map presented in this article shows a somewhat different pattern of malaria risk than the newly produced global *P. falciparum* map from the Malaria Atlas Project which estimated prevalence in children aged 2 to 10 years [39]. Notably, we estimated a higher prevalence of malaria in most areas of the country. Another map produced at a regional scale for West and Central Africa also estimated much lower prevalence (<20%) [40] than that predicted by our model. This regional map was based on surveys conducted in less than 375 locations over all of West Africa, however, these discrepancies may also be explained by additional factors. These two maps were based on historical data, comprising multiple surveys from different seasons with non-standardized data collection protocols and overlapping age groups. Importantly, we chose to predict malaria prevalence for the month of August, which corresponds to the beginning of the season of high malaria transmission, whereas the other maps produced either yearly average or average over the high transmission season.

Malaria parasitaemia risk has been shown to be spatially correlated over distances of about 9 km, a relatively short distance given that all households within a community were geo-referenced to the same location resulting in the loss of fine-scale heterogeneity in malaria risk. The highest risks were found in rural areas, while the lowest risk was in the urban areas (proxied by population density). Although the geo-statistical model’s predictions proved to be relatively accurate, our prediction’s RMSE was of 0.19. Some of the models’ errors in the prevalence estimation could be due to important variables that were not accounted for in our risk maps such as coverage of indoor residual spraying (IRS) and ITN, and distribution and distances to mosquito breeding sites [41]. Unfortunately, no recent entomological studies were carried out in Burkina Faso that could provide more information on mosquito breeding sites and the vector layer of the country’s main rivers and water bodies might have been too coarse to accurately represent smaller breeding habitats. Finally, the absence of information on coverage of malaria control interventions (e.g., IRS and ITN) has prevented the inclusion of related variables in the analysis.

The Burkinabé Ministry of Health set out the ambitious target to reduce the morbidity of malaria by 75% in 2015, using 2010 as a baseline, in its new ‘National Control Program on Malaria from 2011 to 2015’ [5]. The government aims to reach these targets by a series of prevention, treatment and support strategies. Prevention strategies include the two most important vector control measures: IRS, and long-lasting insecticidal net use (LLIN). IRS is recommended for populations in the regions of ‘Sud-Ouest’, ‘Cascades’, ‘Hauts-bassins’, and ‘Boucle du Mouhoun’ (see Additional file 4: Figure S3 for a map of administrative regions). Larval source management is also considered for the mostly urban regions of ‘Centrale’ and ‘Hauts Bassins’. Treatment strategies include early and adequate treatment of malaria with ACT and the prompt treatment of severe malaria cases in reference centers. Support strategies include better information, education and communication (IEC) campaigns and monitoring and evaluation of these programs [5]. Our malaria risk maps indicate that the first step of the target, i.e. IRS in the four regions mentioned above and larval control in two densely populated regions of ‘Central’ and ‘Hauts Bassins’ correspond to priority areas where interventions targeting malaria are more urgently required. However, given our estimated prevalence in the ‘North’ region, targeting hotspots in this area with LLIN and possibly IRS should also be considered.

This study has some limitations. First, because the survey does not differentiate between symptomatic and asymptomatic cases, our maps indicate where parasitaemia detected through microscopy was highest but not necessarily where treatment is most needed. The nature of the data also imposed important limitations in that individual households were not geolocated and only the centroid of the community was mapped. Further, to insure data confidentiality, random spatial error was added to the geographic locations of each cluster: 0.90 km on average for urban clusters and 2.26 km for the rural ones [42]. This random error could attenuate the strength of association between the climatic/environmental variables and parasitaemia risk by introducing some non-differential misclassification of exposures. The strengths of this study lie in its large sample size, rigorous variable selection, and the use of appropriate multilevel and geo-statistical models. An important advantage of Bayesian geostatistical analysis is that it takes into account the spatial correlation inherent to malaria transmission and properly models the effects of the covariates.

## Conclusion

National prevalence of malaria in children under five years of age in Burkina Faso was one of the highest ever recorded by DHS surveys. High spatial variation in malaria risk was exhibited and children living in densely populated areas had the lowest risk of infection. The number of parasitemic children was, however, highest in such urban areas. The determinants of malaria infection described in this study as well as the maps of parasitaemia risk and number of infected children could be used by managers of malaria control programs to define priority intervention areas. Even though the environment plays an important role in malaria transmission, increasing coverage of malaria interventions and disease knowledge but also, improving socio-economic conditions will be key to achieving sustainable malaria burden decreases in Burkina Faso.

## Electronic supplementary material

Additional file 1: **Text S1.** Technical information related to the performed Bayesian Variable Selection and to the type of prior distributions used for fitting the multilevel and geo-statistical models. (DOCX 28 KB)

Additional file 2: Figure S1: Map of surveyed clustered and observed prevalence of malaria parasitaemia in 540 clusters in Burkina Faso (2010). (JPEG 1 MB)

Additional file 3: Figure S2: Maps of the posterior predictive distribution of the 2.5^th^ and 97.5^th^ percentiles of malaria parasitaemia risk in children under 5 years in Burkina Faso for August 2010. (JPEG 1 MB)

Additional file 4: Figure S3: Map of the thirteen administrative regions of Burkina Faso. (JPEG 1 MB)
