# Supplementary material for: Multilevel and geo-statistical modeling of malaria risk in children of Burkina Faso
Source: Parasit Vectors. 2014 Jul 29;7:350. doi: 10.1186/1756-3305-7-350 (PMC4262087; doi:10.1186/1756-3305-7-350)
Supplement: Supplementary file 1 — Additional file 1: Text S1. Technical information related to the performed Bayesian Variable Selection and to the type of prior distributions used for fitting the multilevel and geo-statistical models. (DOCX 28 KB) [file 13071_2014_1628_MOESM1_ESM.docx]

**Additional File 1: Text S1**

**Technical information related to the performed Bayesian Variable Selection and to the type of prior distributions used for fitting the multilevel and geo-statistical models**

Sekou Samadoulougou^1*^, Mathieu Maheu-Giroux^2^, Fati Kirakoya-Samadoulougou^1^, Mathilde M De Keukeleire^1,3^, Marcia C Castro^2^, and Annie Robert^1^

^1^Pôle Epidémiologie et Biostatistique (EPID), Institut de Recherche Expérimentale et Clinique (IREC), Faculté de Santé Publique (FSP), Université catholique de Louvain (UCL), Clos Chapelle-aux-champs 30, bte B1.30.13, 1200 Bruxelles, Belgium.

^2^Department of Global Health & Population, Harvard School of Public Health, Boston MA, USA

^3^Georges Lemaitre Center for Earth and Climate Research, Earth and Life Institute (ELI), Université Catholique de Louvain (UCL), Louvain-la-Neuve, Belgique.

*Corresponding Author (Phone: 32-2-764.32.65, Fax: 32-2-764.33.28).

**Introduction**

The aim of this technical appendix is to provide the reader with further information on the Bayesian Variable Selection using Spike-and-Slab priors and the prior distributions used in fitting the multilevel and geo-statistical models through Integrated Nested Laplace Approximation (INLA).

**Bayesian Variable Selection**

In order to build parsimonious and well identifiable models, Bayesian variable selection was performed [1]. Specifically, we used a Gibbs variable selection algorithm [2] that used spike and slab priors on the model’s coefficients [3]. For the variable’s coefficient *k* in the vector *β,* the spike and slab prior is given by:

*β_k_* ~ *Normal*(0, σ*_k_*^2^)

σ*_k_*^2^ ~ *I*_k_Γ^-1^(a,b) + (1-*I*_k_)*υ_0_*Γ^-1^(a,b)

*I_k_* ~ *Bernoulli*(π*_k_*)

π*_k_* ~ *Beta*(1,1)

The hyperparameters (a, b) of the inverse Gamma were fixed to a=5 and b=25, and υ_0_=0.00025, as proposed by Scheipl et al [4]. In addition, many of our variables were either categorical or polynomials. To enable simultaneous selection or deselection of block of coefficients pertaining to the same variable, we used parameter expansion on these scaled normal mixture of inverse Gamma [4] to avoid oversampling block of coefficients around zero [5]. This prior takes the following form for category *h* of variable *β_k_*:

*β_kh_* = *ϕ_k_ξ_kh_*

*ξ_kh_ ~ Normal*(*m_kh_*, 1)

*m_kh_ = γ_hk_* – (1- *γ_kh_*)

*γ_kh_ ~ Bernoulli*(0.5)

where *ϕ_k_* is given a spike and slab prior, as described above. The best set of predictors was identified using a model with independent random effect at the community level. Model selection was performed using Markov Chain Monte Carlo (MCMC) simulations implemented in JAGS [6]. An adaptive phase of 5,000 iterations and 5 chains of 35,000 iterations were used after discarding 5,000 iterations per chain as burn-in (inference was thus based on 150,000 iterations). The ‘rjags’ and ‘CODA’ libraries were used to run JAGS within the R statistical software [7] .

**Prior distributions of the multilevel and geo-statistical models**

The Bayesian model formulations described in the manuscript was completed using non-informative priors on all model hyperparameters and parameters. Specifically, all regression parameters were assumed to have Gaussian distributions with mean of zero and precision of 0.001. Priors for the hyperparameters of the random effect at the community level were defined on the logarithmic scale. That is, we assumed that the precision of the random effect followed a logGamma(shape=1, scale=1e-5) distribution. For the spatially-structured random effect of the geo-statistical model, the hyperparameters are the precision and spatial scale parameter (κ) of the Matérn model. Again, both hyperparameters are defined on the logarithmic scale and are given vague log-Normal prior distributions, where the prior medians for the hyperparameter are chosen heuristically to match the domain’s spatial scale (see Lindgren 2012 for more information – [8])

**Additional References**

1. Spiegelhalter DJ, Best NG, Carlin BP, Linde AVD: **Bayesian measures of model complexity and fit.** *Journal of the Royal Statistical Society: Series B( Statistical Methodology)* 2002, **64:** 583.

2. Dellaportas P FJaNI: **On Bayesian Model andVariable Selection Using MCMC.** *Statistics and Computing* 2014, **12:** 27-36.

3. Ishwaran H, Rao JS: **Spike and slab variable selection: Frequentist and Bayesian strategies.** *Ann Statist* 2005, **33:** 730-773.

4. Scheipl F, Fahrmeira L, Kneib T: **Posterior consistency of Bayesian quantile regression based on the misspecified asymmetric Laplace density.** *Journal of the American Statistical Association* 2012, **107:** 1518-1532.

5. Gelman A vDAHZBJ: **Using redundant parameters to fit hierarchical models.** *J Comput Graph Statist* 2008, **17:** 95-122.

6. Plummer M: **JAGS: A Program for Analysis of Bayesian Graphical Models Using Gibbs Sampling.** 2013:1-10.

7. Plummer M. rjags: Bayesian graphical models using MCMC. R package version 3-12. <http://CRAN.R-project.org/package=rjags> . 2014.

8. Lindgren F. **Continuous domain spatial models in R-INLA.** The ISBA bulletin. <http://bayesian.org/bulletin> , 14-20. 2012.
