# Supplementary figures and images for: Multilevel and geo-statistical modeling of malaria risk in children of Burkina Faso
Source: Parasit Vectors. 2014 Jul 29;7:350. doi: 10.1186/1756-3305-7-350 (PMC4262087; doi:10.1186/1756-3305-7-350)

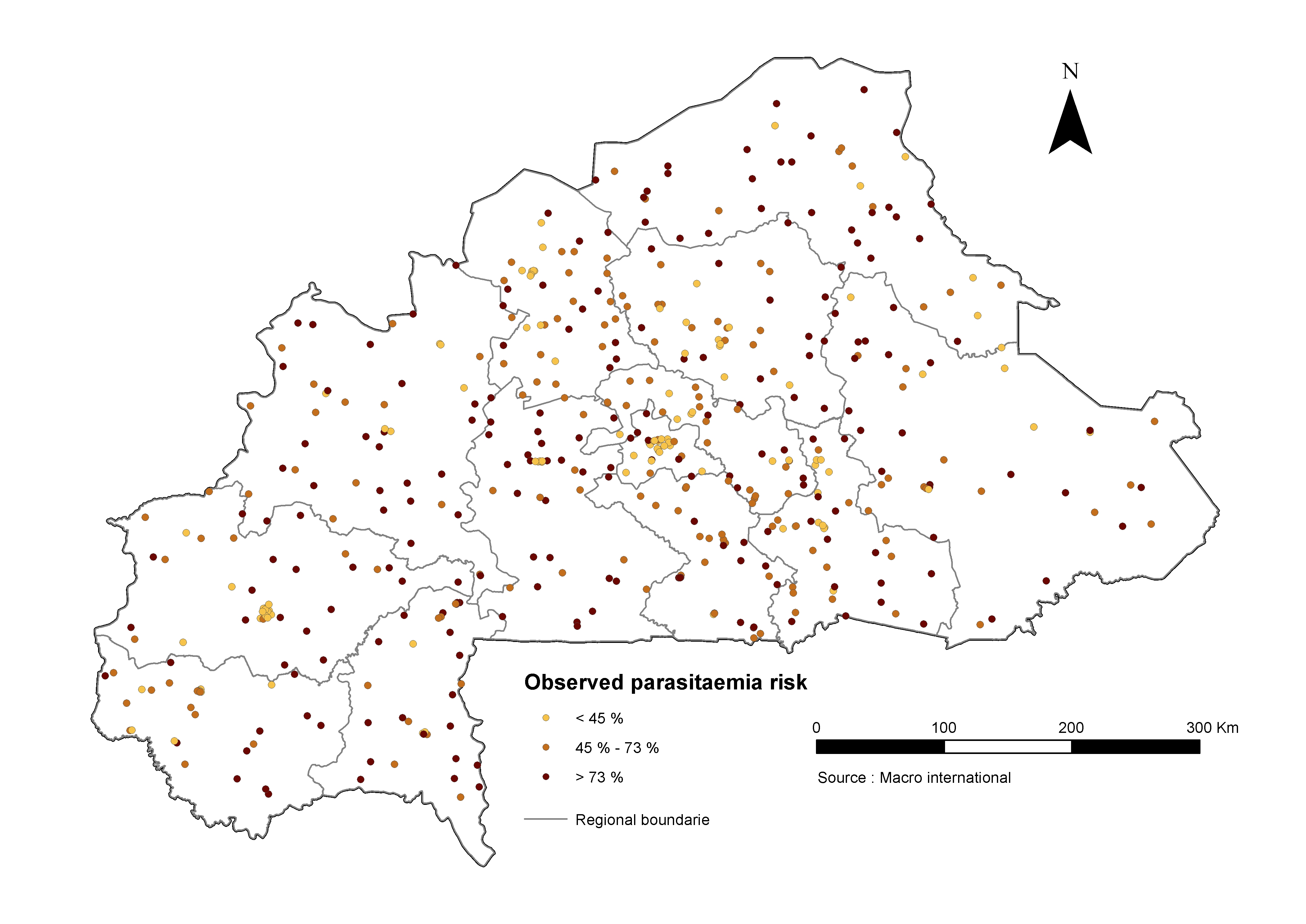

Supplement: Supplementary file 2 — Additional file 2: Figure S1: Map of surveyed clustered and observed prevalence of malaria parasitaemia in 540 clusters in Burkina Faso (2010). (JPEG 1 MB) [file 13071_2014_1628_MOESM2_ESM.jpeg]

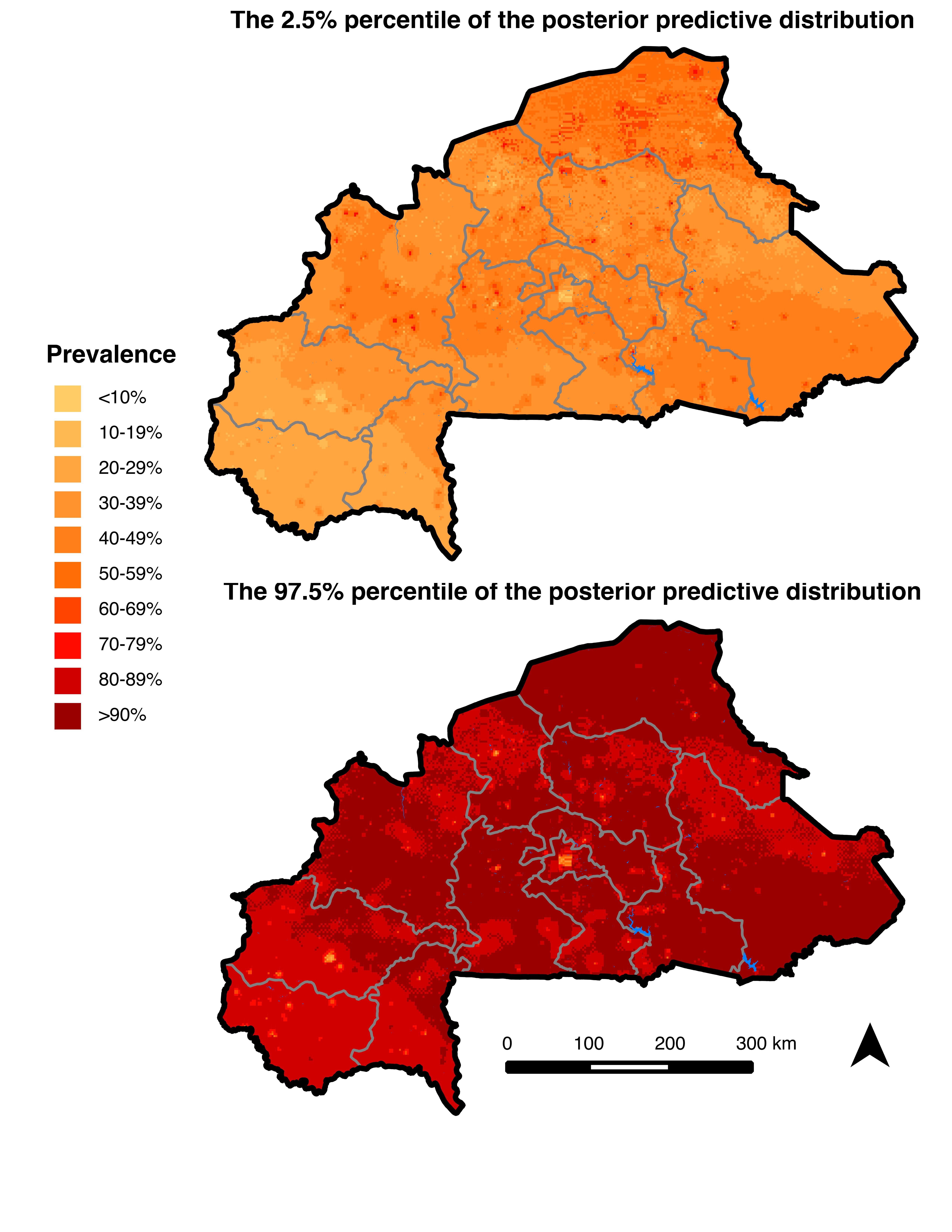

Supplement: Supplementary file 3 — Additional file 3: Figure S2: Maps of the posterior predictive distribution of the 2.5th and 97.5th percentiles of malaria parasitaemia risk in children under 5 years in Burkina Faso for August 2010. (JPEG 1 MB) [file 13071_2014_1628_MOESM3_ESM.jpeg]

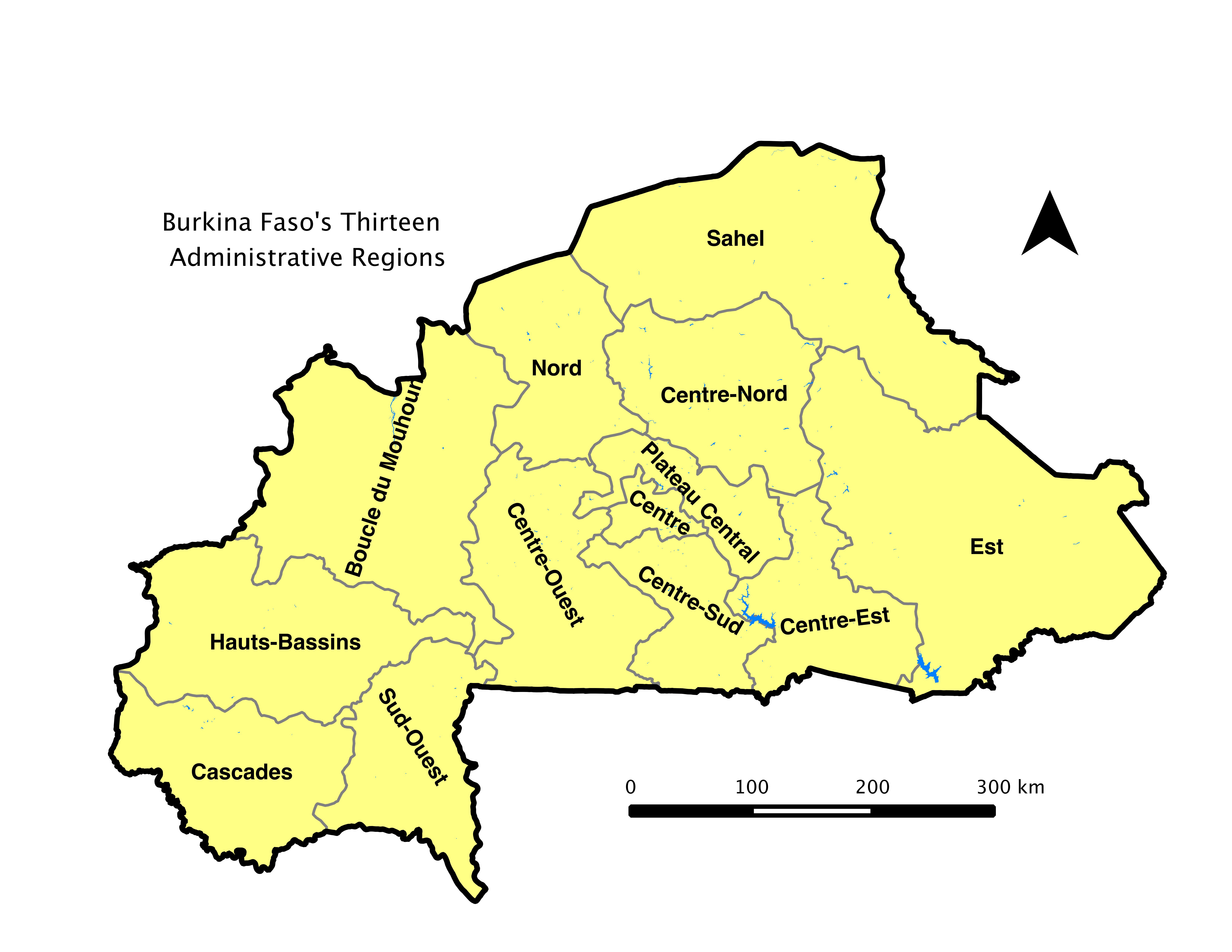

Supplement: Supplementary file 4 — Additional file 4: Figure S3: Map of the thirteen administrative regions of Burkina Faso. (JPEG 1 MB) [file 13071_2014_1628_MOESM4_ESM.jpeg]
